# Combination therapies for the treatment of recurrent central giant cell lesion in the maxilla: a case report

**DOI:** 10.1186/s13256-016-1173-3

**Published:** 2017-03-20

**Authors:** Jefferson Paulo de Oliveira, Fernanda Olivete, Naylin Danyele de Oliveira, Allan Fernando Giovanini, João César Zielak, Leandro Klüppel, Rafaela Scariot

**Affiliations:** Universidade Positivo, 5300 Prof. Pedro Viriato Parigot de Souza St., Campo Comprido, Curitiba, PR 81280-330 Brazil

**Keywords:** Central giant cell lesion, Combination therapies, Treatment, Diagnosis

## Abstract

**Background:**

Central giant cell lesion is a non-neoplastic proliferation, usually asymptomatic, of unknown etiology. The purpose of this case report is to report the diagnosis and the treatment of a recurrent central giant cell lesion in the maxilla.

**Case presentation:**

A 31-year-old Brazilian woman presented to our Surgery Service for evaluation of a cystic lesion in her teeth 13 and 15, although she had previously received endodontic treatment for her teeth 13 and 15 without regression of the lesion. On clinical examination, an increase and painless swelling was observed in her right jaw. An excisional biopsy of the lesion was performed under general anesthesia; the material was sent for pathological examination and a diagnosis compatible with central giant cell lesion was made. She presented again, 10 months after the removal of the lesion, with a recurrent lesion that surrounded her incisors, canine, and right premolar. We suggested that she underwent treatment with intralesional corticosteroids injection. The lesion was significantly reduced and the remainder of the lesion was enucleated. She is monitored at 3-month intervals; at 6 months postoperatively there has been no recurrence.

**Conclusions:**

Central giant cell lesion can have a high degree of invasiveness, which increases the importance of early diagnosis. Combination therapies can provide a favorable prognosis. Periodic monitoring is recommended, thus avoiding the chance of a relapse.

## Background

Central giant cell lesion (CGCL) is a benign proliferative and asymptomatic intraosseous lesion. It is more common in females, in the third decade, without predilection for race [1–3]. It accounts for approximately 7% of all benign lesions in the jaw. These lesions occur more frequently in the mandible than in the maxilla, and occur more frequently on the right side than on the left side [4, 3]. In the maxilla, the lesion may invade the maxillary sinus floor, the orbit and/or nasal passages, which can lead to facial asymmetry, nasal deviation, and mobility of associated dental elements. CGCLs can be classified as aggressive or non-aggressive. Clinical, radiographic, and histological characteristics differentiate their level of aggressiveness and guide the treatment plan [1, 2, 5]. In image examinations, CGCL appears as a variation of small apical lesions to large radiolucent areas, which can be unilocular or multilocular, with a slight opacification within the lesion [1, 6]. On histological examination, CGCL consists of cellular fibrous tissue with multiple foci of hemorrhage, multinucleated giant cell aggregates and, occasionally, trabecular bone tissue [4, 7]. The treatment can be either conservative, with periodic corticosteroids infiltrations, or surgical, by enucleation or surgical resection. Although surgical treatment temporarily eliminates the lesion, clinicians need to be aware of its aggression and morbidity and the risk of relapse [8]. The objective of this case report is to describe the treatment of a recurrent CGCL in the maxilla, with the combination of different therapies based on clinical, radiographic, and histological characteristics.

## Case presentation

**Fig. 1:**
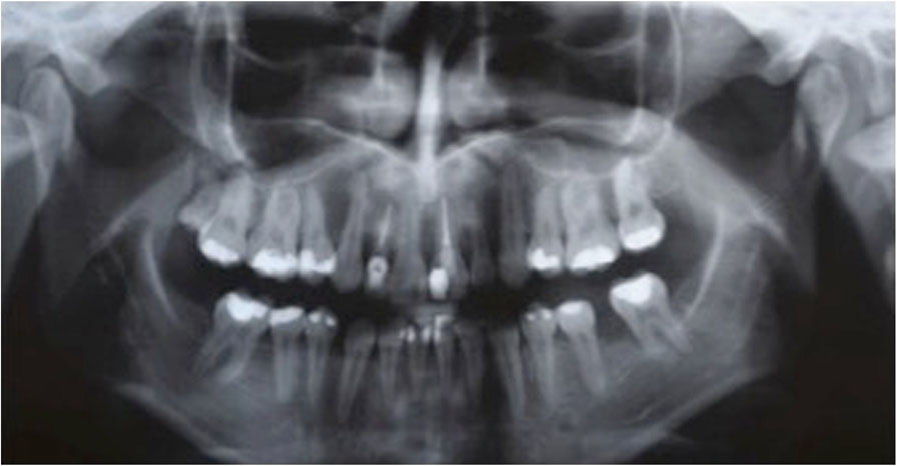
Panoramic radiograph pre-treatment endodontic

**Fig. 2:**
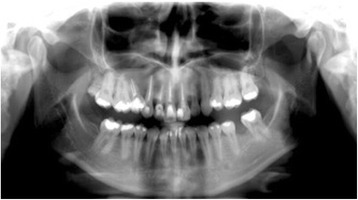
Panoramic radiograph showing a radiolucent lesion associated with teeth 13 and 15

A 31-year-old Brazilian woman presented to our Surgery Department of Oral and Maxillofacial Surgery for evaluation of cystic lesions in her teeth 13 and 15 and a swelling on her right maxilla (Fig. 1). In her anamnesis, she reported that she had already had endodontic treatment for her teeth 13 and 15 without regression of the lesion. Her teeth 14 and 24 had been previously removed for orthodontic treatment. A radiographic and computed tomography (CT) examination showed a radiolucent lesion, diffuse, of approximately 20 mm, involving the teeth 13 and 15. The initial diagnosis was inflammatory periapical cyst (Fig. 2).

**Fig. 3:**
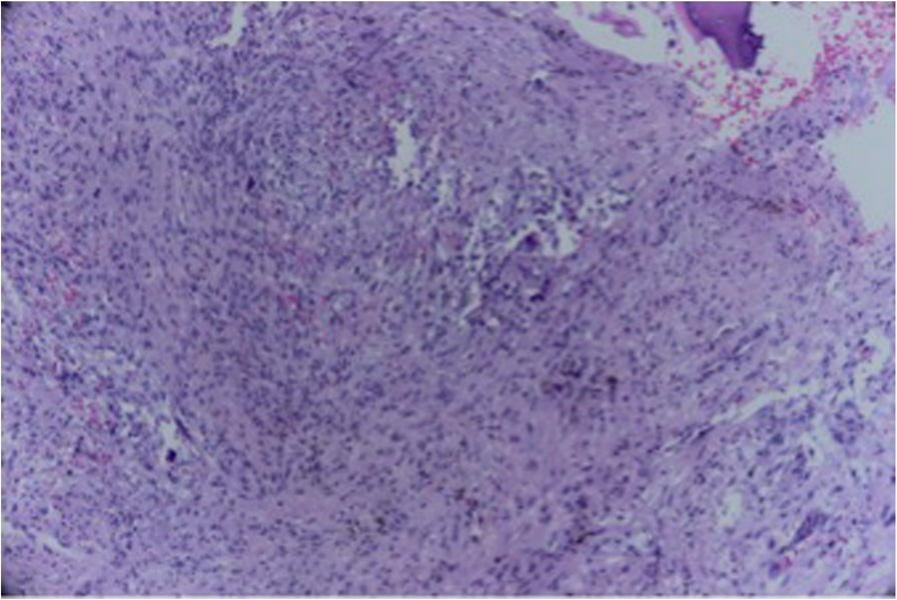
Intra-bone dense connective tissue with oval spindle cells and giant cells, indicating a fibroma (granuloma) central giant cell. Hematoxylin and eosin 40×

**Fig. 4:**
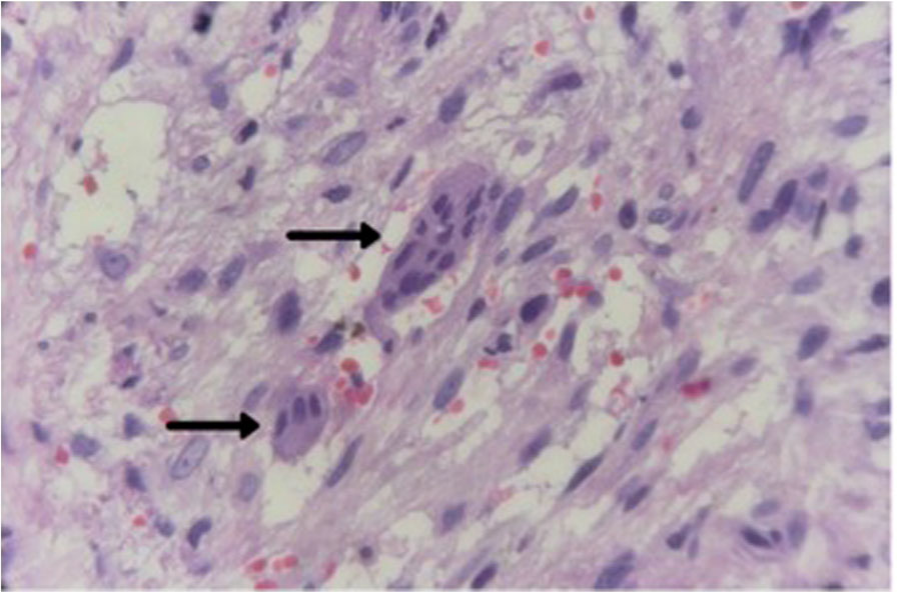
Microscopical details of the biopsied fragment where multinucleated giant cells (*arrows*) surrounded by intense connective tissue and some spindle mesenchymal cells, characterizing the classical histopathological frame of central giant cell lesions, can be found

She underwent preoperative laboratory tests and an excisional biopsy of the lesion was undertaken under general anesthesia. The choice of the use general anesthesia was due to the size of the lesion and possibility of enucleation without removal of the teeth involved. The collected material was sent for pathological examination. The result was compatible with CGCL (Figs. 3 and 4), which differed from our diagnostic hypothesis. After the definitive diagnosis, she was advised to carry out regular monitoring because of the risk of recurrence.

**Fig. 5:**
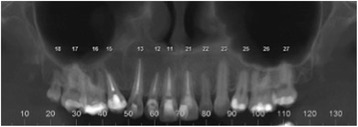
Panoramic image of computed tomography scan showing recurrence

She self-monitored for a period of 10 months; she then presented again with a complaint of swelling on the site, but without the presence of pain. A new CT scan was requested and indicated recurrence of the lesion, which now involved the region’s incisor, canine, and premolar (Fig. 5). New laboratory tests were carried out: alkaline phosphatase, calcium, phosphorus, and parathyroid hormone (PTH). We ruled out the possibility of the lesion being compatible with Brown tumor of hyperparathyroidism. A possible loss of the four dental elements involved was not ruled out.

**Fig. 6:**
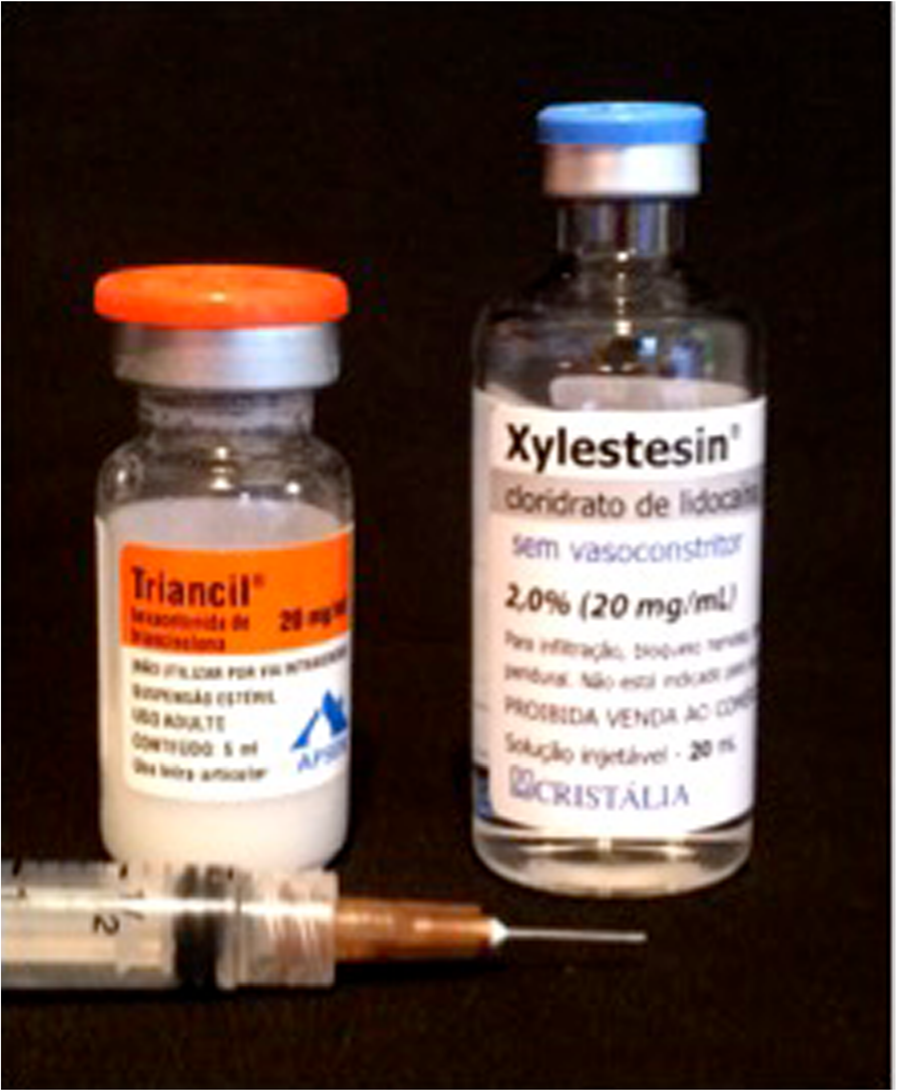
Combination of Triancil® (triamcinolone hexacetonide) and Xylestesin® (lidocaine hydrochloride) used in infiltrations

**Fig. 7:**
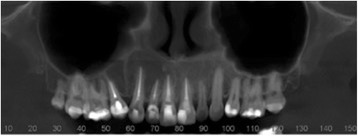
Panoramic image of computed tomography scan showing formation of a bony septum indicating new bone formation

**Fig. 8:**
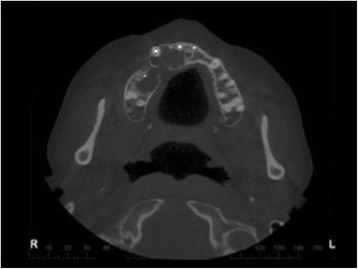
Axial image from computed tomography scan showing lesion divided by bony septum

We proposed new treatment with intralesional corticosteroid injections to our patient in an attempt to reduce the lesion for a future enucleation. She was given a drug application schedule: nine applications in the affected regions with intervals of 15 days between applications. Infiltrations were composed of: Triancil® (triamcinolone hexacetonide) + Xylestesin® (lidocaine hydrochloride; Fig. 6). On average, 1.5 ml of the solution per session was applied at different points. After 5 months, there was a reduction in the lesion. New imaging tests showed that her tooth 16 had external root reabsorption and we decided to carry out an extraction of this tooth during the enucleation of the remaining lesion. We also found the formation of a septum in the damaged area, indicating bone formation (Figs. 7 and 8).

**Fig. 9:**
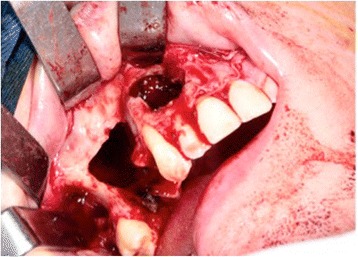
Transoperative image of enucleation of the lesion under general anesthesia, with removal of teeth 15 and 16

**Fig. 10:**
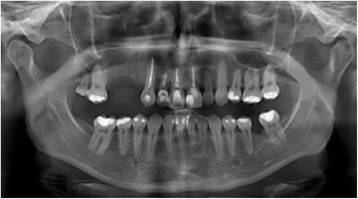
Panoramic radiograph 7 days postoperatively

The enucleation of the remaining lesion was performed under general anesthesia. During surgery we removed tooth 16 as planned. Tooth 15 had no support because the buccal bone plate had been compromised, so we decided to extract it during surgery (Fig. 9). A radiograph was performed 7 days postoperatively (Fig. 10).

**Fig. 11:**
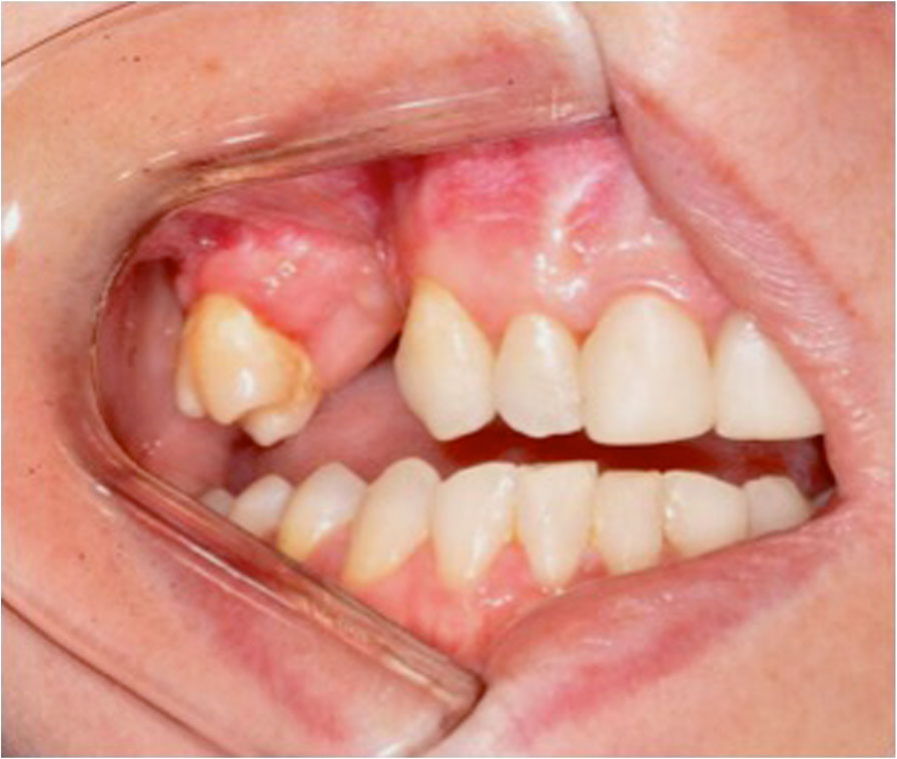
Intraoral photograph at 6 months postoperatively

**Fig. 12:**
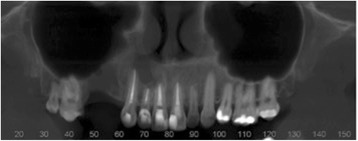
Panoramic image from computed tomography scan showing opacification in the damaged area, indicating bone repair

At 6 months postoperatively, she is on a quarterly follow-up, without recurrence of the lesion (Figs. 11 and 12). A bone reconstruction of the region is planned for 1 year postoperatively.

## Discussion

CGCL is a non-neoplastic proliferation with variable clinical behavior and unknown etiology. However, multinucleated giant cells in this type of lesion are concentrated in areas of hemorrhage and adjacent to blood vessels, suggesting that CGCL represents a phagocytic response to the hemorrhage, indicating that factors such as trauma and extractions may be related to this pathology. CGCL accounts for approximately 7% of all benign lesions in the maxilla. These lesions occur more frequently in the mandible than in the maxilla with more involvement in the right side [4, 2, 5, 9, 10]. CGCL is more common in females, with a ratio 2:1 [7]. This lesion can occur at all ages, but most cases were diagnosed between the second and third decade of life [1, 9, 11].

CGCL can be aggressive and non-aggressive. The non-aggressive form is characterized by slow growth, is typically asymptomatic, and does not pierce cortical bone or induce root reabsorption; it has a low recurrence rate. The aggressive form is characterized by episodes of nonspecific pain, rapid growth, large lesions (>5 cm), paresthesia, root reabsorption, cortical perforation, a high rate of post-treatment enucleation recurrence and often produces edema [12].

On radiographic examination, CGCL varies from small apical lesions to large lesions involving multilocular radiolucent areas of maxilla [1]. The presence of a thin opacification within the lesion is the most significant radiographic signal associated with CGCL [8]. Its appearance is usually consistent with a unilocular or multilocular radiolucency, well or poorly defined, plus a varied expansion and destruction of the cortical plate. This pattern is not pathognomonic radiographically and can be confused with many other injuries in the maxilla and mandible [13]. CT is an excellent imaging examination to demonstrate bone destruction or bone thinning. CGCL located at the apical side or at the roots of teeth can be easily confused with odontogenic inflammatory lesions such as inflammatory periapical cysts or radicular cysts. The common occurrence of these lesions leads the dentist to a definitive diagnosis without major additional tests. CGCL should be included in the differential diagnosis when there is associated periapical radiolucency [14].

On histological examination, CGCL are represented by multinucleated giant cells in a prominent fibrous stroma. Osteoclasts have irregular distribution and are associated with areas of hemorrhage. Structurally the proliferative cells include spindle-shaped fibroblasts, myofibroblasts, and mononuclear inflammatory cells [15]. Foci of hemorrhage with release of hemosiderin pigment are often seen. Immunohistochemical studies in cases of CGCL have helped establish the lineage and pattern of these cells; however, they cannot predict the aggressiveness of the lesion. The final diagnosis possibly rests on the histopathological data because the clinical and radiographic features are nonspecific [13, 7, 11]. The differential diagnosis includes aneurysmal bone cyst, giant cell tumor, and brown tumor of hyperparathyroidism [15].

The traditional treatment of CGCL is surgical excision, enucleation, or *en bloc* resection. This choice depends on factors such as aggressive and non-aggressive form, location, size, and radiographic appearance. Other treatments include radiation, systemic injections of calcitonin, interferon, and intralesional injections with corticosteroids [15]. The approach is calcitocina enhances and inhibits osteoclast activity such as surgery and application of calcitonin [16]. However, due to their great discomfort and relatively long treatment time, this treatment is not well accepted by all patients [2]. Interferon-alpha is useful in managing aggressive CGCL due to its anti-angiogenic effects [17].

Intralesional injections with corticosteroids are increasingly used clinically, and some studies show excellent results. They can be considered a first treatment option. With a less invasive approach, these injections can be used individually or in combination with other therapies, such as surgery and calcitonin [1, 10]. Intralesional injection is preferred than systemic injection, because in first one it is possible to achives a high drug concentration in tissue [12]. Systemic complications associated with administration of corticosteroids are seldom reported [1].

The most aggressive types of lesions require a more radical approach. The management of these lesions depends on clinical and radiographic findings. In general, the enucleation of well-defined and localized lesions is associated with a low recurrence rate. In extensive lesions, based on imaging tests, where there has been cortical drilling, a more radical excision is mandatory [17]. Enucleation remains the most common treatment modality for CGCL; however, a rate of 24% recurrence was reported in non-aggressive lesions, so the preference for associations with other modalities is common [3]. In some cases, loss of teeth and loss of erupted and non erupted teeh are inevitable, as well root reabsorptions in the affected area [15]. Periodic monitoring with radiographs and clinical evaluations should be conducted to prevent recurrence.

## Conclusions

Given the above, the correct diagnosis of CGCL and its degree of aggressiveness is achieved through an analysis of clinical, radiographic, and pathological examinations. The treatment plan may vary from noninvasive therapies, such as medication, to surgical approach, wherein enucleation is proposed. There is also the possibility of a combination of techniques. The combination of two different therapies can be a good solution, since in some cases a single surgical approach leads to facial mutilation.
